# Descending the sanitation ladder in urban Uganda: evidence from Kampala Slums

**DOI:** 10.1186/1471-2458-14-624

**Published:** 2014-06-19

**Authors:** Japheth Kwiringira, Peter Atekyereza, Charles Niwagaba, Isabel Günther

**Affiliations:** 1 Department of Sociology and Anthropology, School of Social Sciences, College of Humanities and Social Sciences (CHUSS), Makerere University, P.O. Box 7062, Kampala, Uganda; 2 Department of Sociology, Kyambogo University, P.O. Box 1 Kyambogo, Kampala, Uganda; 3 Department of Civil and Environmental Engineering, College of Engineering, Design Art and Technology (CEDAT), Makerere University, P.O. Box 7062, Kampala, Uganda; 4 Swiss Federal Institute of Technology –Zurich (ETH-Z) and Centre for Development and Cooperation (NADEL), Zürich, Switzerland

**Keywords:** Sanitation ladder, Improved sanitation, Unimproved sanitation, Latrine use, Cleaning, Open defecation, Maintenance, Kampala, Slums

## Abstract

**Background:**

While the sanitation ladder is useful in analysing progressive improvements in sanitation, studies in Uganda have not indicated the sanitation barriers faced by the urban poor. There are various challenges in shared latrine use, cleaning and maintenance. Results from Kampala city indicate that, failure to clean and maintain sanitation infrastructure can lead to a reversal of the potential benefits that come with various sanitation facilities.

**Methods:**

A cross sectional qualitative study was conducted between March and May 2013. Data were collected through 18 focus group discussions (FGDs) held separately; one with women, men and youth respectively. We also used pictorial methods; in addition, 16 key informant interviews were conducted. Data were analysed using content thematic approach. Relevant quotations per thematic area were identified and have been used in the presentation of the results.

**Results:**

Whether a shared sanitation facility was improved or not, it was abandoned once it was not properly used and cleaned. The problem of using shared latrines began with the lack of proper latrine training when people do not know how to squat on the latrine hole. The constrained access and security concerns, obscure paths that were filthy especially at night, lack of light in the latrine cubicle, raised latrines sometimes up to two metres above the ground, coupled with lack of cleaning and emptying the shared facilities only made a bad situation worse. In this way, open defecation gradually substituted use of the available sanitation facilities. This paper argues that, filthy latrines have the same net effect as crude open defection.

**Conclusion:**

Whereas most sanitation campaigns are geared towards provision of improved sanitation infrastructure, these findings show that mere provision of infrastructure (improved or not) without adequate emphasis on proper use, cleaning and maintenance triggers an involuntary descent off the sanitation ladder. Understanding this reversal movement is critical in sustainable sanitation services and should be a concern for all actors.

## Background

Nearly 800 million people in urban areas worldwide lack access to adequate sanitation. By 2020, nearly 60 percent of Africa’s population will be in urban areas, and within 20 years, the population of most African cities will have doubled [1]. In Sub-Saharan Africa, urbanization has become virtually synonymous with slum growth, with the world’s highest annual slum and urban growth rates almost identical at 4.53% and 4.58%, respectively [2,3]. In contrast to previous attitudes towards slums that characterized them as illegal settlements to be eradicated, slums are now viewed as an inevitable ‘growth pain’ of economic development [4]. In this regard, the United Nations developed a *‘slum target’* (Goal 7, Target 11) of the Millennium Development Goals (MDGs) to improve the lives of 100 million slum dwellers by 2020. However, while monitoring this goal, UN Habitat uses the following criteria: (1) improved access to water and sanitation; (2) improved structural quality of housing; (3) reduced overcrowding; and (4) improved security of tenure [1]. This schema makes sanitation monitoring inadequate because UN-Habitat uses criteria without mention of cleaning and maintenance of the available sanitation facilities.

Sanitation generally refers to the provision of facilities and services for the safe disposal of human urine and feaces. Studies have also shown that improvements in sanitation are not only linked to major health advances but should also have positive indirect effects on several other MDGs, in particular those involving the environment, education, gender equality, and the reduction of child mortality and poverty. The extent to which latrines deliver the intended health benefits depends on how they are used. If latrines are properly used; not soiled, regularly and well cleaned, well covered and emptied in a timely fashion, and if hands are washed after use, latrines deliver enormous health benefits [3,5,6].

In Uganda, slum settlements are characterized by extreme poverty, lack of property tenure, lack of services and infrastructure and an informal economy [3,7,8]. There has been failure by urban local authorities to enforce development control and to provide effective municipal services due to corruption, low revenue collections and poor civic competence among the population [3,7,8]. In most cases, shared human excreta facilities provide an uncertain degree of improvement in sanitation [9,10]. A household is considered to have adequate access to sanitation if an excreta disposal system, either in the form of a private toilet or a toilet shared with a *reasonable* number of people, is available to household members [11]. In 2004, WHO and UNICEF categorized shared private toilets and latrines as ‘improved shared’ under certain conditions such as the facility being located within the dwelling unit, yard or compound [12]. However, the shared facilities were re-designated as ‘unimproved sanitation’ i.e. not hygienically separating human excreta from human contact in recognition of poor operation, many users, lack of maintenance and abuse [1,2,5]. Although the UN-Joint Monitoring Programme (UN-JMP) has made strides in monitoring progress toward the MDG target for sanitation through increased access; no commensurate effort has been made to measure and document the status of sanitation facilities after commissioning –i.e. beyond their physical availability.

**Figure 1:**
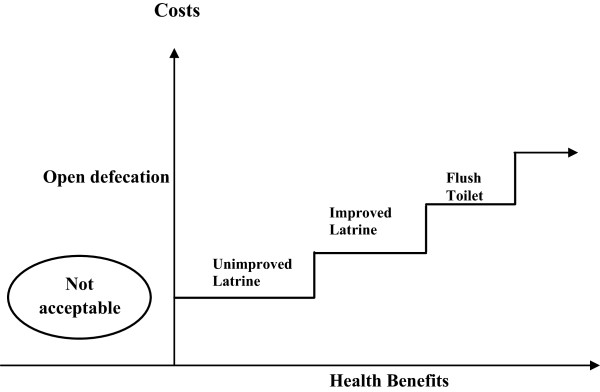
The sanitation ladder.

According to the sanitation ladder^a^, the physical availability of sanitation facilities reflects progress in sanitation. Available literature shows different types of sanitation as rungs on a ladder, with each rung having a higher investment cost and greater health benefits than the one below [13,14]. The bottom of the ladder is open defecation, a practice harmful to health. The first rung is unimproved latrines, which comprise various kinds of pits that vary greatly in their efficacy but provide at best, only basic sanitary protection. The next rung is improved latrines, including a variety of engineered facilities such as San Plat, Ventilated Improved Pit (VIP) latrines and basic pits with slabs. When appropriately used, these facilities provide adequate sanitary protection at reasonable cost. The final rung of the ladder is the flush toilet, which may be connected to either a septic tank or the sewerage network (where it exists). From a health perspective, the most critical movement is from no service (open defecation) or unimproved service (unimproved latrine) to an improved sanitary facility [11] see Figure 1. In between open defecation and the flush toilet are a number of many latrine options ranging from unimproved to improved facilities.

### Source: adapted from [13]

However; the sanitation ladder does not guarantee proper use and the associated health benefits. In Kampala, majority (70%) of the urban poor use shared latrines; with less than half (47%) of the latrines clean enough to be used and another 45% of the facilities being abandoned [9]. The various sanitation initiatives in urban poor areas have not emphasized improved use, cleaning and maintenance of the available facilities; emphasis seems to be on mere sanitation infrastructure [3-5,15]. The purpose of this paper is to show that, the misuse and abandonment of latrines is usually a gradual process that causes descending the sanitation ladder. The infamous ‘descending,’ back to open defecation, is in form of having filthy and dysfunctional latrine structures.

## Methods

The study used a cross sectional qualitative study design. This design was deemed appropriate to facilitate an in-depth understanding of sanitation in slum conditions especially the poor use, lack of cleaning and the gradual abandonment of the available sanitation facilities. Data were collected from six slums in Kampala city, that exhibited low socio-economic indices including; high average latrine user density, a high number of shared latrines, a high percentage of latrines in a zone that were full, low average incomes, big average household sizes, poor latrine cleanliness, low education levels among residents, diarrhoea among children and a low average asset index [9]. After selecting the study zones, we used Google earth maps to divide each of the selected zones into 2 relatively equal parts so as to have 2 different starting points for data collection. The purpose of these starting points and the use of maps were to minimize overlaps and exclusion given the unplanned and congested habitations. Primary data were collected through Focus Group discussions, observation; community transects and Key informant interviews. Three FGDs were conducted per zone (1 for adult males, 1 for adult females and 1 for youth) giving a total of 18 FGDs. FGD participants had to have been resident in the zone for at least 5 years (see Table 1 for other respondent characteristics).

**Table 1:** Typical characteristics of shared latrine users

**Characteristic**	**Percentages**
Proportion of respondents who were household heads	48.21
Tribes of respondents	
Muganda	57.28
Musoga	9.08
Mukiga/Munyankole/	9.31
Mutoro	3.82
Atesot	3.82
Other tribes	16.69
Mean age	33.17
Education level	
No education	19.09
Primary level	44.87
Secondary level	26.01
Tertiary	10.03
Main source of income	
Casual laborer	8.59
Small scale business	45.11
Medium business	5.49
Formal employment	15.51
Dependence on relatives	21.48
Rent collections	2.15
Pension or retired	1.67
Household average monthly income (UGX)	79,792.3
Proportion (%) of respondents who own the houses they stay in	26.49

After prospective group discussion members gathered at the venue, the moderator introduced the topic in order to guide the discussions [16-19]. Local council leaders identified venues such as an open space or rooms from where we conducted the FGDs. The first author usually attended the initial part of the FGD sessions but mainly interviewed key informants. The main issues for discussion were; access to shared latrines, use of latrines and cleaning. To address the likelihood of inhibition, female FGDs were conducted by female researchers and those for males were conducted by male researchers. In addition, training of research assistants on techniques of data collection including use of probes helped to make FGDs an effective approach for data collection. When other community members showed interest in joining the FGD once the maximum number of 10 had been obtained we did not allow them to join the FGD. We told them to wait, after which we briefly told them about the purpose of the study. All the same, we heard their concerns and understanding of shared latrine use, cleaning and maintenance. The views from these spontaneous discussions are not included in this paper owing to the large numbers of people in these discussions, the limited time for discussion and difficulties of writing detailed notes. On average, key informant interviews lasted 45 minutes while FGDs took 60–90 minutes. The observation checklist included the state of different sanitation facilities; hence pictures of shared latrines were taken. Secondary data were obtained through a review of urban sanitation literature from the developing world.

### Data management and analysis

We analysed data manually using content thematic approach. We followed a frame work advanced by Graneheim and Lundman [20] to identify manifest and latent content in the discussion and interview scripts [21]. The first author read FGD and interview scripts several times independently to identify emerging themes and sub-themes. Joint discussions with research assistants were held to compare the identified themes and sub-themes; a process that led to development of a unified list of codes for use in data analysis. The major themes identified were; the mere presence of latrine structures did not mean that they are being used, once people doubted the integrity of a structure they shunned it, the condition of a structure over shadowed its intended use, wet seasons flooded some latrines, smelling latrines repelled prospective users, latrine disuse was disguised open defecation and varying cultures affected the use of latrines. These themes and subthemes were used to code data from focus group discussions and key informant interviews. Sub-group analysis was done, which involved examining the themes and sub-themes in relation to various categories of FGDs (men, women and youth) and key informants. We identified verbatim quotations which have been used in presentation of study findings.

Secondary literature was thematically analysed using content analysis. This approach involved identification of the study themes and sub-themes following multiple reading of interview and discussion manuscripts.

### Quality assurance

Before the study, research assistants were taken through a rigorous training session as well as a pre-test session and then paired during data collection and continuously assessed. Research assistants were fluent in English and the main local languages used in the city (Luganda, Swahili and Runyakitara) and had experience working in urban poor settings. To ensure completeness and correctness of data, after each day the research team converged for review of the day’s activities where data was cleaned and verified before storage and processing. Both manual and electronic backups were used.

### Ethical considerations

The study was approved by the Research and Higher Degrees Committee for the School of Social Sciences, College of Humanities and Social Sciences; Makerere University which considered all technical and ethical issues. Clearance was also obtained from the local leaders in the respective slum zones of Kampala City. An introductory letter issued by Makerere University was presented to local leaders in addition to explaining the purpose of the study, confidentiality, voluntary participation; anonymity and freedom to withdrawal from the study were clearly explained [22]. Verbal consent to participate in the study was obtained from all study participants. Participants were free to withdraw from the study if they felt uncomfortable. No persons lacking capacity to consent were enrolled or involved for the study [23]. In addition, study participants’ identifiers were not recorded. The need for confidentiality was emphasized during training of research assistants prior to data collection [24,25]. With the study findings being published, this shall reduce further resource wastage, for instance; by not conducting other studies in the same area without first benefiting from these findings. Publishing and information sharing minimizes community research fatigue and wastage of valuable resources [26]. In this way, the beneficence and equity principles were upheld, with the respondent recruitment process and research practice being non-coercive and confidential. The conceptualisation, design, execution and reporting of study findings complies with the Relevance, Appropriateness, Transparency of Procedure and Soundness of Interpretive approach (RATS) guidelines for qualitative research as supplied by Biomedical Central Journals.

## Results

This section presents study findings for each rung on the sanitation ladder. Findings indicate that whether sanitation facilities were improved or not; once not properly used and cleaned, latrines fail in their basic function of providing sanitary protection. The study shows that, while unimproved sanitation facilities pose structural challenges in use and cleaning, improved facilities on the other hand fail to serve their purpose when misused or not properly cleaned. Female FGD participants indicated that poor access and many users led to the misuse and poor cleaning and discouraged latrine use. Women further indicated that cleaning challenges were experienced once a latrine stance was shared by more than five households. This also led to waiting for long especially at peak hours of morning and evening. Because of the delays, some people resorted to unhygienic methods of human excreta disposal especially the use of polythene bags and containers. This finding is consistent with other studies on slum sanitation [8,10,11].

All data sources (FGD, Key informant, observational and pictorial) showed that unimproved latrines were more misused than the improved types especially due to uncooperative user practices and the difficulties involved in their cleaning. This constraint in user cooperation led some users (especially women and children) to shun the filthy facilities on account of the potential health risks. Key informants asserted that latrines were not emptied due to the high cost of emptying associated with difficulties of access, in addition to the low incomes among the users. Pit emptying was also avoided for fear of collapse since the pits were not lined. On the other hand, improved latrines suffered from cleaning challenges that were not structural, but related to many users amidst poor access control and supervision. Users of shared improved facilities reported the misuse of collected levies by caretakers; a practice that discouraged further payments. Findings indicated two forms of open defecation; the ‘overt’ type of directly defecting on the ground, and the ‘covert’ type where people defecated in polythene bags and littered the surrounding with feaces. The most commonly mentioned barriers to latrine use and motivation for open defecation were; access difficulties such as security for women and children especially at night coupled with long distances, a facility being locked, steep inclines before access; dirt and filth in the facility, many users (that also occasioned long waiting durations) high pit filling rates; expensive and complicated pit emptying; tenant perceptions and attitudes to latrine use and cleaning; payment for latrine facilities; the lack of privacy for females especially during menstruation; impacts of flooding due to high water table on latrine cleanliness and inappropriate waste disposal in pits.

Generally, improved latrines were cleaner than the unimproved latrines, this is in agreement with other studies on sanitation in slums [27,28]. The few functional water borne facilities were being used by few people especially landlords. Water borne facilities that had many users had long been abandoned due to misuse and poor maintenance. For water born borne facilities, the main cause of disuse and abandoning related to the lack of constant water supply and the absence of consumables especially toilet paper, soap and brushes for scrubbing. This is in agreement with other studies in urban poor areas [14,29]. For all the shared latrine categories, there were users who simply lacked a sense of responsibility to clean the latrine that they used. Ironically, the improved facilities that were usually locked, also suffered an environmental cost when their surroundings were fouled overnight. Some users preferred not to use these latrines in resentment of the dirty environment. Many and uncoordinated users were a cause for quick filling, fouling and subsequent abandoning;

“We manage to keep our toilet clean because everyone has a key and we are few users. The challenge is that when you move out of the house there are many people who do not use latrines and end up defecating in the open when it is dark. This is a big challenge for us who try to use our toilet properly; we do not see much benefit since we still encounter feaces in our neighbourhood.” Landlord Kisenyi Zone

Even when latrine facilities are available, users especially women and children complain that they are not conveniently located, that they are unclean, or that using them at night poses a security risk. Children, women, the elderly and the disabled were especially at greater risk. Similar findings have been reported in Indian, Peruvian and African cities [30-33]. The fear to use poorly maintained facilities related to the fear of falling in the pit, getting infections and the general unfavourable access due to high rise constructions (see Additional file 1: Photo 1) and being locked at some times of the day and for most of the night time.

“The latrine we use is sometimes locked by the owner. We have to ask for the key from his house all the time. The latrine is even far from our house with a very steep ladder. We also fear going out in the night when everyone has slept. In the morning, you find dumped polythene bags in the neighbourhood as well as feaces around the latrine.” Tenant Gogonya Zone

The latrine facility in Additional file 1: Photo 1 shows the challenges of access for children, women, the elderly and the physically disabled.

Latrine cleaning habits:

“People are different and behave differently. You will find that even when there are materials and water for cleaning, some people will not bother to clean.” Land lady Kisasizi Zone

Large families sometimes find difficulty in renting houses:

“Tenants with bigger families often have difficulty in getting accommodation easily because landlords here fear that large families lead to the latrines filling quickly. Such tenants subsequently devise means of easing themselves and in most cases resort to open defecation.” Local leader Kisenyi 1 Zone

Landlord’ attitude to latrine use:

“Some landlords complain when one takes long in the latrine or makes many visits in a day to the facility. These landlords argue that the latrine will not serve its intended life span which would make them incur unforeseen costs.” Tenant Gogonya Zone

Payment for latrine facilities:

There are clean toilets in our neighbourhood but one has to pay and yet you cannot start paying for the toilet before knowing what the children will eat that day!” Tenant White Nile Zone

Observation data indicated that many latrines were not well maintained. Shared latrines were characterized by a repugnant smell which discouraged their use, even when they were clean. Such obnoxious facilities left the user equally smelling. One female in Dobbi zone shared her dilemma as she contemplates latrine use in view of the social cost. She said thus;

“When it starts raining, you can only use that latrine when you are sure that you must take a shower; if you do not shower, no one can stand you… Every time you leave the latrine, the people you meet can tell where you have been. That is why I prefer using the polythene bag at home.”

The problem of smell was especially pronounced during the rainy season which was further encouraged by the convenience of disposing-off human waste in drainage channels and compounds. Latrine disuse was also occasioned by uncooperative user practices which led some users to shun the filthy facilities (see Additional file 1: Photo 2).

“Most slum dwellers do not seem to mind dirty latrines probably because they already face many unfavourable conditions. People look at water as consumption and therefore critical for survival with no substitutes; and yet latrines have many substitutes -people feel everybody needs water, while not everybody needs a latrine since there are many ways of defecating.” KCCA Health official

Facilities like the one shown in: Additional file 1: Photo 2 drive females away since women would prefer to use clean facilities especially during menstruation when they need privacy and utmost hygiene to avoid contracting urinary tract infections (UTIs). Other sanitation studies indicate similar concerns with a typical gender divide [34-36].

Female needs:

“Naturally, women are sanitation conscious and if they cannot find a hygienic latrine they will rather use none at all. Unlike men, when women squat they risk catching infections which makes them mind the status of the facility more than men.” KCCA health official

Landlord attitude:

“For us, nothing is being done, our landlord is not doing anything and he does not mind. Ours is full and one would not want to look there.” Female tenant Gogonya

Wet seasons and their effect:

“Ours is a flooding latrine and yet children play around it when maggots are moving around. The issue of toilets in this area is terrible; when entering you don’t want to breathe or touch anything. After you have managed to use it you do not want to think about it again.” Youth tenant Gogonya Zone

Inappropriate waste disposal and lack of enforcement:

“Some people look at latrines as waste disposal pits for all manner of waste including, pads, broken glasses, old clothes, pampers and all that is unwanted. This practice complicates use, cleaning and maintenance. Because of this, latrines attract no respect.” Land lord Dobbi Zone

### Indiscipline; people who just don’t care and varying cultures

Some users of shared latrine facilities simply passed off as ‘undisciplined’ and ‘careless’ in disrespect of authority or any form of shared resource. Interviews with landlords showed that these were the greatest cause of open defecation and the use of ‘flying toilets’. This echoes the ‘tragedy of the commons’ where users lack a sense of responsibility, sustainability and restraint that also sows the seeds of free riding that eventually makes public and shared goods challenging to maintain [37-39]. Therefore, once the role of improved access, proper use and cleaning is not well understood, the consequence is usually latrine misuse and subsequent abandoning (see Additional file 1: Photo 3).

Poor cleaning habits and indiscipline

‘You need to stay here and see these women; they deny that their children misuse the latrine. Even when you catch them red-handed they quarrel for almost a week and then resort to using polythene bags because they do not want to participate in cleaning.” Landlady Jjuko zone

“Many of my fellow tenants are very uncooperative. They have a feeling of; ‘let so and so clean since we all use the latrine’; and for me I would not mind cleaning, but no sooner do you finish leaning than the facility is dirtied gain. You reach a point and get fed up with ceaseless cleaning and the equally ceaseless dirtying.” Tenant, Kisenyi zone

“Here, people have a motto of ‘To Whom it May Concern.’ This could be because of many nationalities and tribesthat are so diverse such as Congolese, Somalis, and Rwandese, Sudanese etc. There is very little interest in community affairs.” Local leader Gogonya Zone

Such discourtesy calls for a strict discipline and coordination regimen and other practical measures to punish offenders.

Access to latrines

“When I started locking my latrines, the main challenge was breaking the padlocks at night. I have since put a metallic door that also locks both from outside and inside and now the latrines are clean.” Land lady Kisaasizi Zone

Both male and female FGDs emphasised that excessive use of alcohol was linked to the misuse of shared latrines. Drunkards were reported to break padlocks, misuse and leave the facility dirty. Some female participants claimed that by cleaning a shared latrine one risks acquiring all manner of diseases including skin infections, Ebola, Cholera, dysentery, diarrhoea and possibly syphilis. This perception did not encourage the cleaning of shared facilities and subsequently led to their abandoning.

“In our culture, latrine cleaners have a lot of bad omen. They do not produce, are poor and die early. If you marry one of them, you must produce a latrine cleaner. It is a very risky job!” FGD participant -Congolese

Some slum dwellers held the view that, *‘feaces of children are as harmless as the children themselves’* and therefore do not need to be disposed-off in latrines. This culminates in littering of compounds with feaces of children and in some cases the disposal of these feaces with food leftovers and grey water. Such practices spread diseases on account of cultural misconceptions about feaces of children.

*“You need to see a child’ excreta so as to know about a child’ health: what the child ate can be told from the feaces, whether the child drunk well, whether the child is satisfied or even sick. When I return home, I have to ask where the child defecated once I come back. Because of this reason we do not dispose-off the feaces of children in latrines*.” *Tenant –Mother Gogonya 1 zone*

Key informant and observation data showed that poorly used latrines spread disease carrying germs through various means such as scavengers, rodents, people’ feet and hands including children who play with contaminated polythene bags and by using contaminated rain water from rooftops^b^. This finding is in agreement with other findings from other un-serviced areas in the developing World [25].

## Discussion

When latrines are filthy or locked and users cannot have access, it becomes a beginning point for descending the sanitation ladder. In response, people resort to the use of containers and polythene bags which find their way in the wider environment in the form of flying toilets^c^. Many users mean the associated challenges of keeping the facility clean, quick filling, emptying costs and sometimes the impossibilities of emptying that usually led to abandoning the facility and back to open defecation. It was such and similar challenges that sometimes discouraged landlords to take on tenants with large families. Therefore, a latrine is sanitary and safe (improved) not only because of the technology and material used but also because of good sanitation practices and behaviours among users. An improved latrine that is not correctly used and not emptied still poses high risks of environmental contamination and disease [11,40]. Thus, rolling out a physical investment program without the accompanying promotion of hygiene makes little sense, and yet, too often, these ^‘^*soft’* aspects of sanitation are overlooked, and priority is given to only the hardware. An implication for policy makers and practitioners is that, latrine ‘cleanliness’ should connote a facility being free from vectors and odours such as flies and rodents and with no faecal matter lingering in or around the latrine. This makes having a cover for the drop hole and a proper vent pipe critical or else no impact shall be realised given that flies would still find the feaces and in subtle ways spread pathogens [41-44].

Because there is a general lack of space and an implicit motivation for more rental income than improving the sanitation situation; some landlords rent latrines in the vicinity for their tenants. This worsens the status of such facilities because tenants are never willing to clean such latrines arguing that they pay rent which should take care of the cleaning. This lack of coordination leads to cleaning and maintenance challenges that subsequently drive the-would-be users away from using the poorly kept facilities. In such cases, tenants are left with no option but to use ‘flying toilets’. This mode and status of latrine access and use restricts demand and access to sanitation, coupled with poor enforcement by urban authorities. In some cases, sanitation facilities were hired and temporarily attributed to a rental unit to serve a given purpose or escape closure.

### Getting and staying on the sanitation ladder

In probing what happens once sanitation facilities are misused; their cleaning poor, and use discouraged, the paper shows that the consequences of misuse and non-use push people gradually back to the bottom of the sanitation ladder. Whereas statistics show progress in sanitation by presence of and access to sanitation facilities, this is not adequate. The physical count conceals the contrasting patterns in use and the effects on health and the environment. This study poses the main challenge in slum sanitation as; *how to keep urban poor households up the sanitation ladder once the ascending has started.* Although the costs of meeting the MDG sanitation target are high, so is the associated health dividend [6].

There is need for sustainable actions in sanitation provisions that are neither project bound, nor time specific but actor oriented in relation to; the individual; (male and female), household (culture and orientation), neighbourhood (affordability for operation and maintenance) and local leadership systems and levels (inclusion and ownership). This is in agreement with Household Centred Environmental Sanitation (HECS) which places the household and its neighbourhood at the core of the planning process that enables people to lead healthy and productive lives while the environment in which they live is protected and enhanced [15,44]. The idea of sanitation provisioning and sustainability implies many variables, albeit in different dimensions and magnitudes that encompass; shared values in relation to toilet training norms, local leadership, property ownership, (community) participation, urban planning, regulation and enforcement. There is need for relevant planning, sanctions, coordination and various enforcement mechanisms as well as broader service provision for the urban poor [45,46]. This requires a substantial change in cultural values and behaviour that emphasises personal responsibility and discipline. It is important to remember that safety concerns by some users also drove people away from using unimproved latrines, especially those facilities with make shift structures. Without such initiatives, people may not use latrines at all or they may use them in a way that undermines the potential health benefits as evidence from Kampala slums shows. Clearly, health is one consideration in the demand and use of a latrine, but not necessarily the foremost in people’s minds. This is qualified by the many alternatives to latrine use that had proved convenient and ‘dignified’ even when they posed clear health hazards.

**Figure 2:**
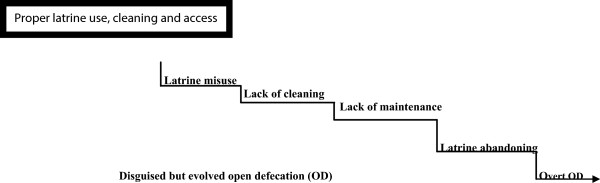
Conceptualising the descent off the sanitation ladder.

In order to break the disease chain, initiatives aiming at achieving the first rung on the sanitation ladder should also aim at ensuring that the latrines are in proper use and clean. Water borne facilities were more of a burden than a solution; due to the income expenditure disconnect which meant water disconnections due to unpaid bills, blockages, staining due to lack of cleaning materials, filth and smell among others. The water borne facilities meant a higher water and maintenance budget that proved expensive and unaffordable to the majority of the slum dwellers.Safe, sure and sustainable sanitation is facilitated by the availability of viable structures for human interface. However, the study shows that sanitation provision begins with availability of structures but goes beyond structures to entail their use, operation and maintenance. The poor status of sanitation facilities is a combination of institutional, structural and individual challenges that equally need a combined solution. The conceptual model presented in Figure 2 argues that, the sanitation ladder and its benefits only apply when people correctly use, clean and have easy access to safe and hygienic latrines.

When a latrine facility suffers misuse and lack of cleaning, the users of such facilities are long off the sanitation ladder in terms of consequences on their health and the wider environment.

The reality of shared latrine facilities in slums has two implications; in one instance, it means that women will strive as much as possible to keep the sanitation facilities clean. On the other hand, women will out rightly shun sanitation facilities that expose them to risks of infections hence resorting to open defecation. Such gendered undertones have been previously documented in an attempt to emphasise equity in sanitation provision and use [37,47]. Because poorly kept latrines have the same effect as open defecation, sanitation provisioning should focus on sustainable demand for hygiene around the person that can only be extended to the sanitation facilities. This is possible through behaviour changes that do not tolerate open defecation in form or essence.

Therefore, whereas the sanitation ladder is a useful tool; monitoring progress towards the sanitation target of the MDGs could be more useful if it can be refined to be based on the functioning of sanitation systems beyond a mere sanitation hierarchy.

Even when insights from this study have implications for sanitation planning in Kampala city given that 60% of Kampala residents reside in slums; these findings should be understood as limited by only using qualitative data. The adopted approach of analysing sanitation through ‘descending the ladder’ has not been previously used, and as such may not get wide reception among traditional sanitation players. Be as it may, we hope this approach shall enable better sanitation provisioning for the urban poor.

## Conclusion

All manner of unsanitary facilities (latrines and toilets) irrespective of being improved or not; deliver the same negative effects as open defecation. Therefore, if not well maintained, fixed point defecation can be no better than crude open defecation. Underlying sustainable sanitation in urban poor settings is the fact that sanitation is about taking good private decisions that impact in a substantive way as a *shared good* in the wider environment for all sharing users. The paper shows for that a wanted latrine is one that is well-constructed, accessible, clean, affordable and well-maintained. This calls for an enforcement framework for compliance in various ways and means. In the absence of this, people will shun or misuse any latrine or toilet for either, explicit or implicit open defecation. Plausible cleaning options for latrines in slums include; the user group (tenants, landlords and other categories of users) hiring a paid cleaner, sharing cleaning on a rotational basis or can incorporate the costs of cleaning in their rental calculations and user fees. Any, or a mix of the preferred cleaning methods should also be highly coordinated and monitored for supervision, enforcement and punishing offenders. Short of this, climbing the sanitation ladder in slums (of Kampala) may prove temporally and reversible given that ‘clean’ is a continuously negotiated process.

### Endnotes

^a^A visual aid representing progress from no sanitation facility (open defecation), the lowest most basic sanitation provision to the best possible facility which is a flush toilet.

^b^Refer to the ‘6Fs’ of feacal oral contamination; Fields, Feaces, Fluids, Food and Fingers.

^c^The indiscriminate disposal of feaces includes; wrapping in polythene bags and then casting them on roof tops at night or dumping them in drainage channels. Sometimes people get hit by these ‘flying’ toilets. Innocent children also play with the polythene bags as balls in which feaces have been wrapped.

## Abbreviations

KCCA: Kampala capital city authority; LC: Local council; GIZ: German technical cooperation; NGO: Non –governmental organisation; FGDs: Focus group discussions; KII: Key informant interview; IDI: In depth interview; UN: United nations; UTI: Urinary tract infections; OD: Open defecation; UN-JMP: Joint monitoring program of the United Nations; CHUSS: College of humanities and social sciences; ETH: Swiss federal institute of technology; EAWAG: Department of water and sanitation in developing countries; NADEL: Centre for development and cooperation; SANDEC: Swiss aquatic institute; VIP: Ventilated improved pit latrine; MGDs: Millennium development goals.

## Competing interests

The authors declare that they have no competing interests.

## Authors’ contributions

All authors JK, PA, CN and IG participated in study design. JK was involved in data collection, analysis and writing the manuscript. Most of the writing was done by JK and comments received from fellow authors. All authors reviewed and approved the manuscript for submission.

## Authors’ information

JK is a PhD Student at Makerere University and Lecturer of Sociology at Kyambogo University, Kampala Uganda; Kampala Uganda; PA is Associate Professor of Sociology at Makerere University, Kampala, Uganda; CN is an Environmental Engineer and Senior Lecturer at Makerere University in the College of Engineering Art and Design (CEDAT); IG is Assistant Professor and Chair of Development Economics at Centre for Development and Cooperation at The Swiss Federal Institute of Technology –Zurich (ETH-Z), Switzerland.

## Pre-publication history

The pre-publication history for this paper can be accessed here:

http://www.biomedcentral.com/1471-2458/14/624/prepub

## Supplementary Material

Additional file 1 Photo file for Descending the sanitation ladder in urban Uganda. Click here for file
