# Supplementary material for: Descending the sanitation ladder in urban Uganda: evidence from Kampala Slums
Source: BMC Public Health. 2014 Jun 19;14:624. doi: 10.1186/1471-2458-14-624 (PMC4071028; doi:10.1186/1471-2458-14-624)
Supplement: Additional file 1 — Photo file for Descending the sanitation ladder in urban Uganda. [file 1471-2458-14-624-S1.docx]

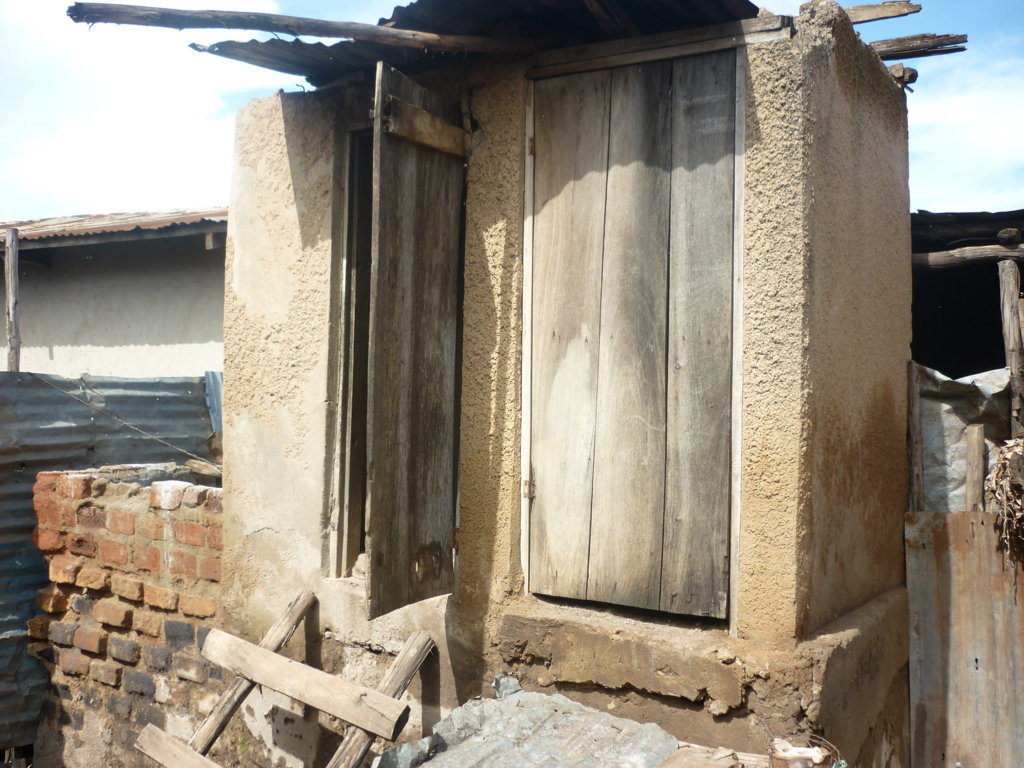


**Photo 1:** Note the steep incline with ‘strip stairs’ raising about 1.5 meters above the ground. This makes it very risky for the elderly, women, children and the disabled.

**Photo 2:** This latrine was also located adjacent to a drainage channel in a high water table area. There is little one can add in describing how repugnant the facility is. Note the filth and wide hole that seems unusable. With such facilities prevalent, when it rains flood water mixes with the contents which spreads diseases just like open defecation.

**Photo 3:** The polythene bags and other litter show the extent of flying toilets, open defecation and the unused latrine facility.
